# Endogenous Curing Mechanism and Self-Healing Properties of an Epoxy Resin (E-51) in Alkaline Environments of Cement-Based Materials

**DOI:** 10.3390/polym18020262

**Published:** 2026-01-18

**Authors:** Qianjin Mao, Yuanlong Wang, Runfeng Li, Yuhuan Zhou, Shuqing Shi, Suping Cui

**Affiliations:** 1 State Key Laboratory of Materials Low-Carbon Recycling, Beijing University of Technology, Beijing 100124, China; 2 College of Materials Science and Engineering, Beijing University of Technology, Beijing 100124, China; 3 Beijing Building Materials Academy of Science Research, Beijing 100041, China

**Keywords:** epoxy resin, alkaline catalysis, endogenous curing, cement-based materials, self-healing concrete

## Abstract

Regarding the issues arising from the addition of external curing agents in the application of epoxy resin in cement-based materials, this paper explores the feasibility of endogenous curing of epoxy resin in the alkaline environment of cement-based systems. It further analyzes and investigates the curing characteristics of epoxy resin without external curing agents and their impact on the performance of cement-based materials. Differential scanning calorimetry, mechanical property testing, microstructural observation, and electrochemical impedance spectroscopy were used to study the mechanism of sodium hydroxide (NaOH) catalyzing the process of bisphenol-A epoxy resin (E-51)-based curing, the influence of moisture and temperature on curing kinetics, and the performance of epoxy resins in mortar and self-healing concrete. The results showed that E-51 achieved self-curing under alkaline conditions in the absence of an external hardener. However, moisture significantly inhibited the reaction process. Elevating the temperature and reducing environmental humidity effectively promoted the curing reaction. In cement-based materials, E-51 exhibited endogenous curing by the inherent alkalinity of the system, remarkably enhancing the compressive strength of mortar. At 60 °C, mortar containing 10% E-51 (by cement mass) exhibited a 1.5-fold higher compressive strength than that of the control group without E-51 at 14 days of curing. It demonstrated higher healing efficiency in a microencapsulated self-healing concrete system than the traditional curing agent systems. Concrete specimens with damage induced by loading at 60% of their compressive strength exhibited 100% recovery of ultrasonic pulse velocity after storing indoors for 28 d. The findings of this study can provide theoretical basis and technical support for the application of epoxy resins in cement-based materials without the need for curing agents.

## 1. Introduction

Cement-based materials, the most widely used construction materials worldwide, are characterized by low tensile strength and high susceptibility to cracking, which significantly reduces the service life of cement-based constructions [1]. Therefore, inhibiting or eliminating microcracks through self-healing mechanisms before crack propagation is important for enhancing concrete durability [2]. Epoxy resins have been used in the modification [3,4] of cement-based composites and designing [5,6] self-healing systems owing to their excellent chemical stability, mechanical properties, and bonding capabilities. They are the core material in microcapsules used for self-healing systems. They can rapidly fill cracks upon capsule rupture owing to their favorable fluidity [7,8], filling cracks with widths of 9–22 µm within 12 h and completely sealing microcracks with a width of 42 µm within 21 d [9]. Liu et al. [10] developed a cement-based composite material with integrated self-healing and -sensing functions by coincorporating carbon nanotube–carbon black and epoxy resin microcapsules into the cement matrix. Epoxy resins can be combined with other materials for fabricating multifunctional self-healing systems [11,12]. However, they should be cured to optimize their performances. A three-dimensional (3D) interpenetrating network is formed during the curing of epoxy resins owing to the formation of chemical bonds with cement, which physically fills the matrix, significantly enhancing the mechanical strength of the composite material [13,14].

The use of traditional epoxy resins relies on externally added curing agents (such as amines), increasing cost and construction complexity. The curing agents might also interfere with cement hydration and adversely affect long-term durability [15]. Thus, self-crosslinking epoxy resins not requiring external curing agents [16] have gained attention. These resins exhibit high-temperature self-curing through nucleophilic ring-opening reactions, achieved by introducing reactive functional groups (such as amide groups) into the epoxy molecular chains. However, the self-curing temperatures of such systems range from 80 °C to 150 °C [17,18], exceeding the typical service temperature range of concrete and limiting their application in conventional structural engineering, particularly for self-healing concrete systems at ambient temperatures. Achieving curing at ambient temperatures while preventing premature reactions within the capsules would open new avenues for the application of epoxy resins in self-healing concrete.

A hydrated pore solution in cement is strongly alkaline (pH = 12–13), providing an endogenous environment for alkali-catalyzed curing of epoxy resins. This condition can be used to achieve autonomous curing by epoxy resins, which can enable the development of epoxy resin-based cement composites in the absence of external curing agents, with simple processing methods and good compatibility. This has the potential for advancing structural repair and intelligent civil engineering materials. One year exposure in indoor and outdoor environments of epoxy resin-modified mortar in the absence of curing agents demonstrated significantly higher flexural and compressive strengths than those containing curing agents, exhibiting up to 1.8- and 1.6-fold (indoor), and 1.7- and 1.5-fold (outdoor) improvement, respectively [19]. This suggests that the presence of epoxy resins in mortar might have undergone self-curing during long-term curing, and the addition of external curing agents might even adversely affect the mechanical performance.

However, current findings remain unclear regarding whether epoxy resins can undergo alkali-catalyzed curing at room temperature. Zhu et al. [20] used nuclear magnetic resonance analysis to demonstrate that a sodium hydroxide (NaOH) aqueous solution could catalyze the ring-opening reaction of epoxy groups, but failed to exhibit significant curing. Yan et al. [21] indicated that even under alkaline and elevated temperature conditions, epoxy resin emulsions without curing agents failed to fully cure within concrete. Ohama [22] reported that although alkali-catalyzed curing of epoxy resin-based cement was limited at room temperature, its strength could be significantly enhanced through hot pressing or steam curing. These discrepancies suggest that owing to multiphase, multiscale complexity of the cementitious matrix and variability of service environments, the curing mechanisms, kinetics, and their influence on the macroscopic performance of epoxy resins in such systems remain unclear. Systematic research on alkali-catalyzed curing behavior of epoxy resins under varying temperature and humidity conditions is notably lacking.

The present study reports the systematic investigation of the curing behavior and mechanisms of bisphenol-A epoxy resin (E-51) under alkaline conditions. Differential scanning calorimetry (DSC) were used to elucidate the reaction kinetics of epoxy groups catalyzed by NaOH, indicating the inhibitory effect of moisture on the curing process. Subsequently, E-51 was incorporated into cement mortar to study the influence of its endogenous curing property on the compressive strength under different temperatures and humidity curing conditions. Furthermore, the micromorphology of epoxy cured inside the cement was observed. Finally, E-51 was encapsulated in capsules to function as a healing agent, which was evaluated for its effectiveness in self-repairing concrete cracks. This study breakthroughs the application limitations of epoxy resins that depend on curing agents by elucidating the catalytic curing mechanism and kinetics of epoxy resins in alkaline environments, providing both theoretical and experimental foundations for the development of performance-controllable epoxy–cement composites.

## 2. Experimental Details

### 2.1. Materials

E-51 was supplied by Sinopec Baling Petrochemical Co., Ltd., Yueyang, China. NaOH was supplied by Tianjin Guangfu Technology Development Co., Ltd., Tianjin, China, Tetraethylenepentamine (TEPA, epoxy curing agent) was supplied by Tianjin Fuchen Chemical Reagent Co., Ltd., Tianjin, China.

Raw materials for epoxy microcapsule self-preparation: sodium alginate was procured from Tianjin Guangfu Fine Chemical Research Institute, epoxy diluent (1,4-butanediol diglycidyl ether) was procured from Tianjin Letai Chemical Co., Ltd., Tianjin, China, sodium dodecylbenzene sulfonate was procured from Tianjin Zhiyuan Chemical Reagent Co., Ltd., Tianjin, China, and anhydrous calcium chloride was obtained from Tianjin Fuchen Chemical Reagent Factory.

Raw materials for mortar preparation: P·I 42.5 Portland cement was supplied by Qufu Zhonglian Cement Co., Ltd., Jining, China. Its primary chemical composition and mineralogical composition are listed in Table 1, and the International Organization for Standardization (ISO) standard sand was supplied by Xiamen ISO Standard Sand Co., Ltd., Xiamen, China, Its particle size distribution is shown in Table 2.

Raw materials for concrete preparation. (i) Cement: P·I 42.5 Portland cement, with its primary chemical and mineral compositions are shown in Table 1. (ii) Fine aggregate: Manufactured sand, with a fineness modulus of 2.6 and grading within Zone II (medium sand) was supplied by Zhucheng Jiusan Building Materials Co., Ltd., Zhucheng, China. (iii) Coarse aggregate: Crushed stone, with a particle size range of 10–20 mm and a crushing value of 11.7% was supplied by Zhucheng Jiusan Building Materials Co., Ltd. (iv) Mineral powder (S95): Primary chemical and mineral compositions are shown in Table 3. It was supplied by Henan Borun Casting Materials Co., Ltd., Gongyi, China. (v) Fly Ash: Primary chemical and mineral compositions are shown in Table 4. It was supplied by Henan Hengyuan New Materials Co., Ltd., Hangzhou, China.

Deionized water was used for all experiments except for concrete tests, for which tap water from Beijing was used.

### 2.2. Sample Preparation

#### 2.2.1. Epoxy–NaOH Blending Systems

E-51 and NaOH powder were uniformly mixed with a mass ratio of 10:1 to obtain the E-51–NaOH-p mixture. The mixture was allowed to stand under specified conditions for a designated period. After the completion of the period, the samples were analyzed via DSC (NETZSCH, Selb, Germany).

E-51 epoxy resin was blended with a NaOH aqueous solution with a mass ratio of E-51:NaOH:H_2_O of 10:1:1. The NaOH solution was first prepared by dissolving NaOH in water with a 1:1 mass ratio. E-51 was mixed homogeneously in the aqueous NaOH solution to form the E-51–NaOH-aq mixture. The prepared samples were analyzed via DSC.

#### 2.2.2. Preparation of Epoxy Capsules

Deionized water (500 mL) and 8.125 g of sodium alginate were added to a three-neck flask. The mixture was stirred in a water bath at 60 °C until sodium alginate was completely dissolved. Subsequently, 0.91 g of sodium dodecylbenzene sulfonate was added to the sodium alginate solution, and stirring was continued to obtain mixture 1. Meanwhile, 60.74 g of E-51 and 2.43 g of diluent were mixed by heating in a 60 °C water bath. The diluted E-51 solution was then added to Mixture 1 and stirred at 60 °C for 1 h to yield Mixture 2. Mixture 2 was extruded using a needle and added dropwise into the CaCl_2_ solution with a concentration of 24 g mL^−1^. After standing for 3 h, the epoxy capsules were filtered, washed three times with anhydrous ethanol and dried at 40 °C.

The prepared epoxy capsules were spherical, with a particle size of 0.7–2.0 mm and a core content of 64%.

#### 2.2.3. Preparation of Mortar Specimens

Following the method of testing cements [determination of strength (ISO) (GB/T 29756-2013)] [23], a mass ratio of cement–sand–E-51–water of 1:3:0.1:0.5 was mixed and homogenized using a mechanical mixer. For mortar specimens containing a curing agent, TEPA was introduced with water at a dosage of 10 wt.% of E-51.

(1) The uniformly mixed mortar was cast into 40 mm × 40 mm × 160 mm prismatic molds. The specimens were cured under designated conditions until the specified ages. Compressive strength tests were performed for the cured samples.
(2) The uniformly mixed mortar sample was poured into cylindrical molds with dimensions of Ø100 mm × 50 mm. The samples were cured under the specified conditions until predetermined ages. The cured samples were analyzed via electrochemical impedance spectroscopy (EIS).

#### 2.2.4. Preparation of Paste Specimens

To prepare paste specimens, the cement-to-water ratio was set at 1:0.5, with E-51 comprising 10% of the cement mass. The prismatic molds used for casting measured 100 mm × 100 mm × 100 mm. After curing under standard conditions (20 °C, relative humidity = 95 ± 5%) for 24 ± 2 h, the specimens were demolded and subsequently placed in a constant-temperature oven at 40 °C until reaching 28 days. The prepared specimens were used to investigate the microstructure of epoxy resin within the cementitious materials.

#### 2.2.5. Preparation of Concrete Specimens

Based on the mixture proportions specified in Table 5, cement, fly ash, ground granulated blast furnace slag, sand, and coarse aggregates were weighed and placed into a concrete mixer, and the dry materials were mixed uniformly. Water was added, and the mixture was blended for 30 s. Subsequently, E-51 capsules were introduced to the mixture, which was stirred until a homogeneous mixture was obtained. The resulting concrete slurry was cast into prismatic molds (100 mm × 100 mm × 100 mm) for molding. After standard curing [20 °C ± 2 °C, relative humidity (RH) = 95% ± 5%] to the designated age, the specimens were preloaded to 60% of their compressive strength to induce the formation of artificial internal cracks. The cracked specimens were allowed to heal naturally at ambient temperature for a prescribed period. The obtained samples were analyzed using the ultrasonic pulse velocity test.

### 2.3. Test Methods

#### 2.3.1. DSC Analysis

DSC analysis was performed using a NETZSCH STA449F3 instrument (NETZSCH, Selb, Germany). The nonisothermal tests were conducted under a nitrogen atmosphere with a flow rate of 50 mL min^−1^ and a heating rate of 5 °C min^−1^. The isothermal tests were performed under the mentioned conditions: a nitrogen atmosphere at a flow rate of 50 mL min^−1^, and a heating rate of 50 °C min^−1^ to reach the target temperatures set at 60 °C, 80 °C, 100 °C, and 120 °C.

#### 2.3.2. EIS Method

EIS measurements were conducted using a PARSTAT 4000+ electrochemical workstation (Princeton Applied Research, Oak Ridge, TN, USA) over a frequency range from 1 Hz to 10^7^ Hz. Considering the significant influence of the sample water content on the EIS results, the samples were immersed in water for 24 h before testing. Moistened filter paper was placed at both ends of the samples during measurement to maintain consistent moisture content [24].

#### 2.3.3. SEM Method

Prepared paste samples, they were crushed into 2–3 mm fragments. Then, the fragments were immersed in acetone for 24 h to remove uncured epoxy resin, after which they were removed and dried in a vacuum oven at 60 °C for 48 h and then coated with a gold layer. Processed samples for observation under a Scanning Electron Microscope (SEM, Zeiss, Oberkochen, Germany, Sigma 300) and analysis by Energy Dispersive Spectroscopy (EDS, Oxford, Abingdon, UK, Xplore 30).

#### 2.3.4. Ultrasonic Testing Method

Ultrasonic testing was conducted using a KON-NM-4A concrete ultrasonic velocity detector (manufactured by Beijing KONCR Engineering Co., Ltd., Beijing, China) to measure the ultrasonic wave velocity of concrete specimens at various curing ages. The ultrasonic transmitter and receiver were positioned at opposite ends of the specimens to determine and record the ultrasonic wave velocities at different ages.

#### 2.3.5. Compressive Strength Testing of the Mortar

The compressive strength of mortar was determined in accordance with the method of testing cements [determination of strength (GB/T 29756-2013, ISO method)]. Measurements were performed using an integrated compression–flexure universal testing machine (Model CDT305-2, Mester Industrial Systems (China) Co., Ltd., Shenzhen, China) on specimens cured to specified ages. Six parallel samples were tested to obtain the average value, with the measurement error controlled within 10%.

## 3. Results and Discussion

### 3.1. Alkali-Catalyzed Curing of E-51

#### 3.1.1. Curing Behavior of E-51 with NaOH at Room Temperature

**Figure 1:**
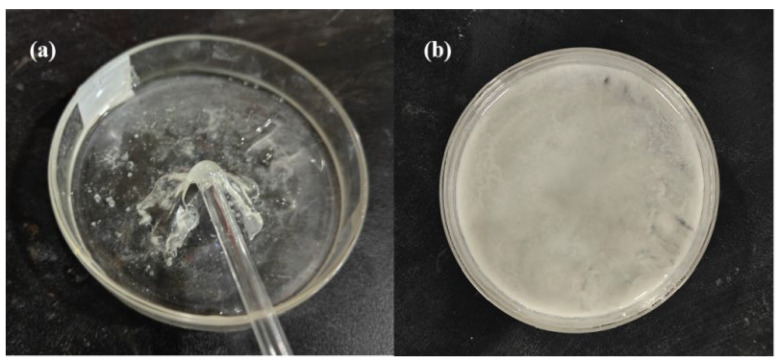
Image of E-51 and NaOH on Day 30 of storage at room temperature: (**a**) E-51–NaOH-p and (**b**) E-51–NaOH-aq.

E-51–NaOH-p and E-51–NaOH-aq mixtures were allowed to stand at room temperature to observe the curing behavior. On Day 30, E-51–NaOH-p is significantly cured, whereas E-51–NaOH-aq is not cured (Figure 1).

With an extended storage time, the curing degree of E-51–NaOH-p gradually increases. On Day 150, the Shore hardness of the cured product is 25 ± 5 HA, which is insoluble in organic solvents, indicating the formation of a 3D crosslinked network. In contrast, E-51–NaOH-aq fails to cure even after storing for 270 d. Thus, E-51 can exhibit curing properties under alkaline conditions at room temperature, albeit with a slow reaction rate and prolonged curing period. These findings are consistent with the reported results [19]. When a substantial amount of water is present in the system, the curing reaction of E-51 is significantly inhibited, which aligns with the reported results [20].

**Figure 2:**
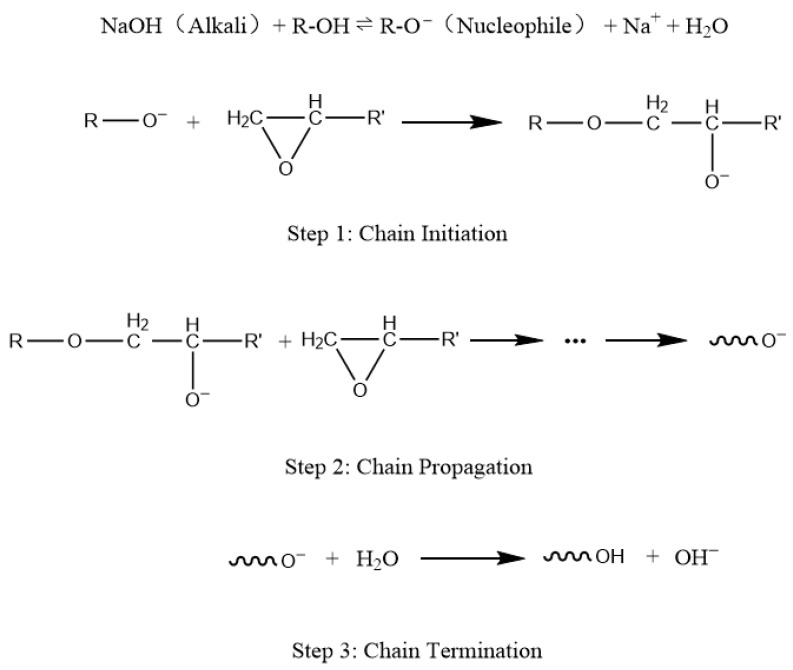
Schematic of the mechanism of base-catalyzed epoxy curing.

This phenomenon of curing in a mixture of E-51–NaOH-p can be explained by the base-catalyzed epoxy resin-based curing. Figure 2 shows that the NaOH base acts as an initiator, first reacting with hydroxyl (–OH) groups in the resin to generate alkoxide anions (RO^−^). These RO^−^ species are strong nucleophiles that attack the epoxy groups, initiating ring-opening polymerization and forming new active sites, enabling chain propagation. As E-51 comprises multiple secondary –OH groups, multiple active centers can be generated, ultimately forming a crosslinked polymer. The active centers might be deactivated by the reaction with water or acidic impurity, causing chain termination.

The reason epoxy groups cannot cure in an aqueous solution system containing NaOH is that water inhibits the generation of RO^−^. In the first step reaction shown in Figure 2, the presence of a large amount of water makes it difficult for the reaction equilibrium to shift to the right. Furthermore, the weak nucleophilicity of OH^−^ and hydroxyl groups is insufficient to initiate the ring-opening of the epoxy group.

#### 3.1.2. Kinetics of Base-Catalyzed Epoxy Curing

**Figure 3:**
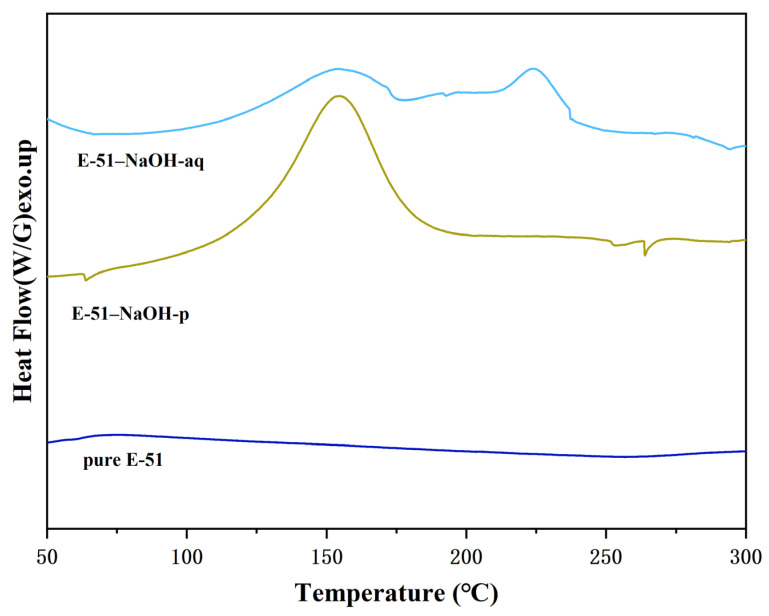
Nonisothermal DSC curves of pure E-51, E-51–NaOH-p, and E-51–NaOH-aq.

Differential scanning calorimetry (DSC) was used to investigate the influence of the physical state of NaOH (solid vs. solution) on the curing kinetics of E-51. The nonisothermal DSC curves of pure E-51, E-51–NaOH-p, and E-51–NaOH-aq are presented in Figure 3.

Figure 3 shows that the DSC curve of pure E-51 is flat with no significant exothermic peak, indicating that the epoxy resin fails to cure spontaneously in the absence of a catalyst. E-51–NaOH-p and E-51–NaOH-aq exhibit distinct exothermic peaks, confirming that the alkali catalyzes the E-51 curing process during heating. Due to the relatively low reaction rate, no notable exothermic signal appears when the temperature is <100 °C. An increase in the temperature to ~110 °C accelerates the reaction, with the appearance of an exothermic peak. Thus, heating significantly promotes base-catalyzed E-51-based curing.

The curve of E-51–NaOH-p presented in Figure 3 displays a single exothermic peak, suggesting that polymerization proceeds continuously after initiation, representing a kinetically controlled single-step process. The broadening of the peak is attributed to the slow reaction rate at low temperatures. The exothermic peak of E-51–NaOH-aq is broad, which is ascribed to the influence of water in the system. Heat is absorbed during water evaporation while simultaneously quenching active centers in the temperature range of 50–100 °C, inhibiting the reaction. Water nearly evaporates at >100 °C, and the reaction is accelerated, although residual water partially suppresses the reaction, which prolongs the curing process.

**Figure 4:**
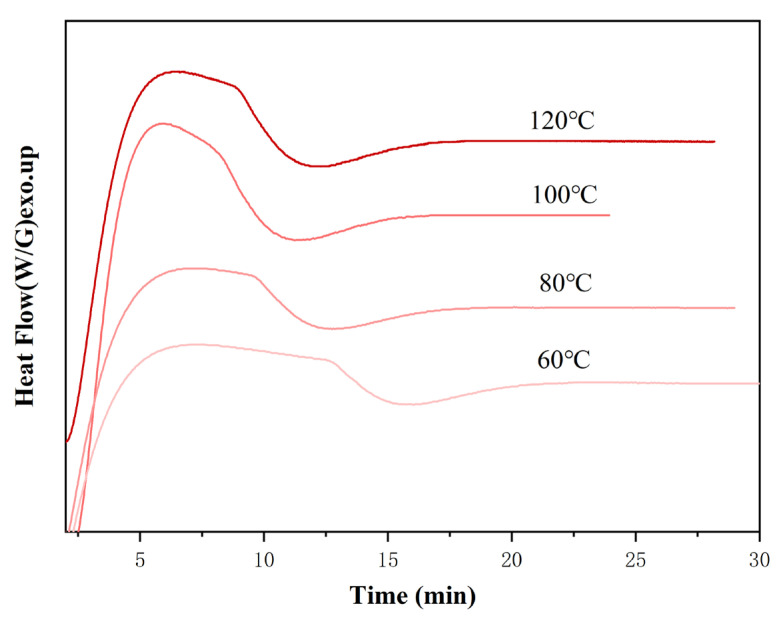
Isothermal DSC curves of E-51–NaOH-p.

The isothermal DSC curves of E-51–NaOH-p at 60 °C, 80 °C, 100 °C, and 120 °C are presented in Figure 4.

Figure 4 shows a distinct exothermic peak at 60 °C. As the temperature increases, the reaction accelerates, the peaks become sharper and higher, and the reaction time shortens from ~16 min at 60 °C to ~11 min at 120 °C. The total heat released at each temperature is similar, indicating that extending the reaction time at low temperatures can also complete E-51 curing.

#### 3.1.3. Quantitative Characterization of the Relative Degree of Epoxy Curing

**Figure 5:**
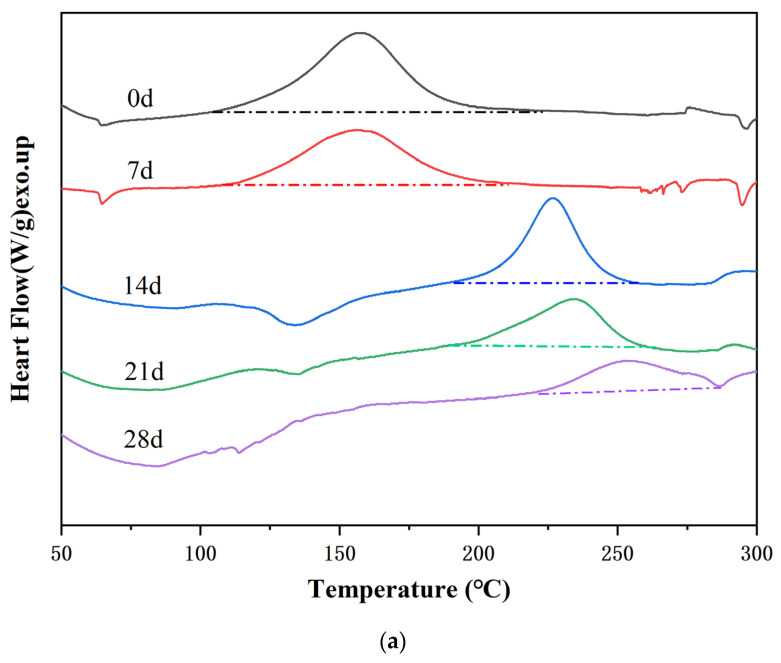

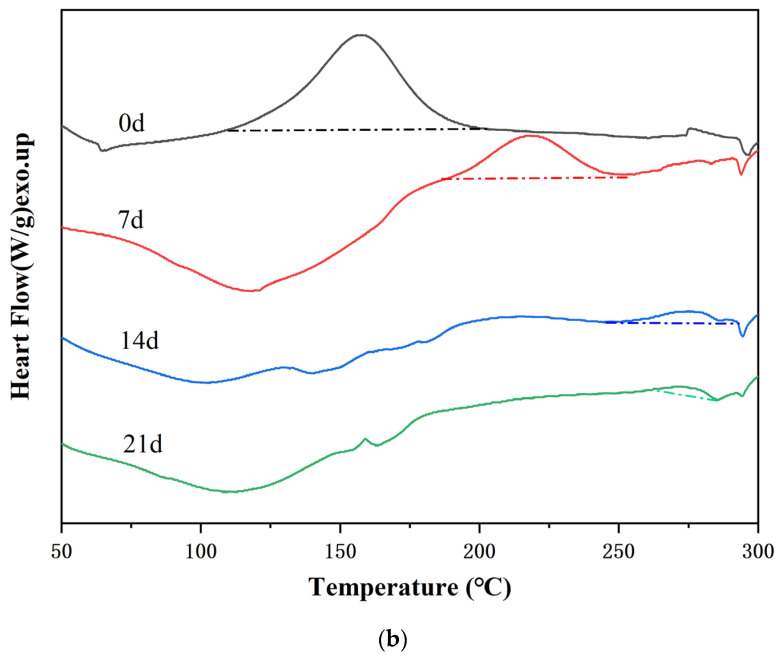
DSC curves of E-51–NaOH-p at different storage times at (**a**) 25 °C and (**b**) 40 °C.

The curing degree of E-51–NaOH-p after storing at room temperature and 40 °C for different durations was quantitatively analyzed via DSC. The DSC curves of the samples stored at room temperature for 0, 7, 14, 21, and 28 d are shown in Figure 5.

Figure 5 shows that the exothermic peak in the DSC curves shifts toward higher temperatures with prolonged storage time. Figure 5a shows that the glass transition temperature of the sample increases from ~60 °C at Days 0 and 7 to ~120 °C at Days 14 and 21, indicating an increase in the degree of curing.

Considering the total reaction enthalpy (Δ*H*_0_) of the sample at Day 0 as the reference, the residual reaction enthalpy (Δ*Hₜ*) at Day *t* was determined. The relative curing degree *α* was calculated using Equation (1).

(1) $$\alpha =\frac{{∆H}_{0\;}-{∆H}_{t}}{\;{∆H}_{0}}=\times \;100$$

The reaction enthalpy values were obtained by integrating the area under the exothermic peaks, and the corresponding relative curing degrees were calculated (Table 6).

Table 6 shows that the curing degree achieved on Day 7 at 40 °C exceeds that achieved on Day 21 at 25 °C. Moreover, the α value of the E-51–NaOH-p is 97.26% on Day 21 of curing at 40 °C. These results demonstrate that temperature significantly accelerates the curing reaction of E-51.

### 3.2. Endogenous Curing of E-51-Added Mortar

Using the alkaline environment of cementitious materials, it is possible to cure epoxy resins without adding external curing agents. We investigated the effects of endogenous epoxy curing on the performance of cement-based composites. E-51 was incorporated into mortar comprising the mentioned components in the mass ratio of cement–sand–water–E-51 of 1:3:0.5:0.1 to form Sample. Two control groups were prepared: Control 1 comprised no E-51, while Control 2 comprised an additional curing agent (TEPA) with 10 wt.% of E-51. Specimens were cured under the mentioned conditions.

C1: immersed in water at 25 °C.
C2: 25 °C and 95 ± 5% RH.
C3: 25 °C and 55 ± 5% RH.
C4: an oven at 40 °C.
C5: an oven at 60 °C.

#### 3.2.1. Effect of Endogenous Epoxy Curing on Compressive Strength of Mortar

**Figure 6:**
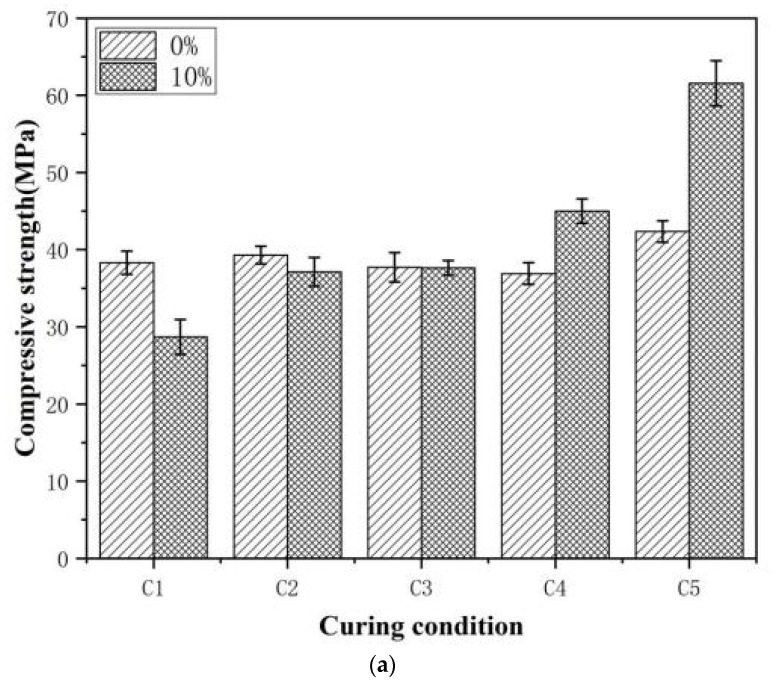

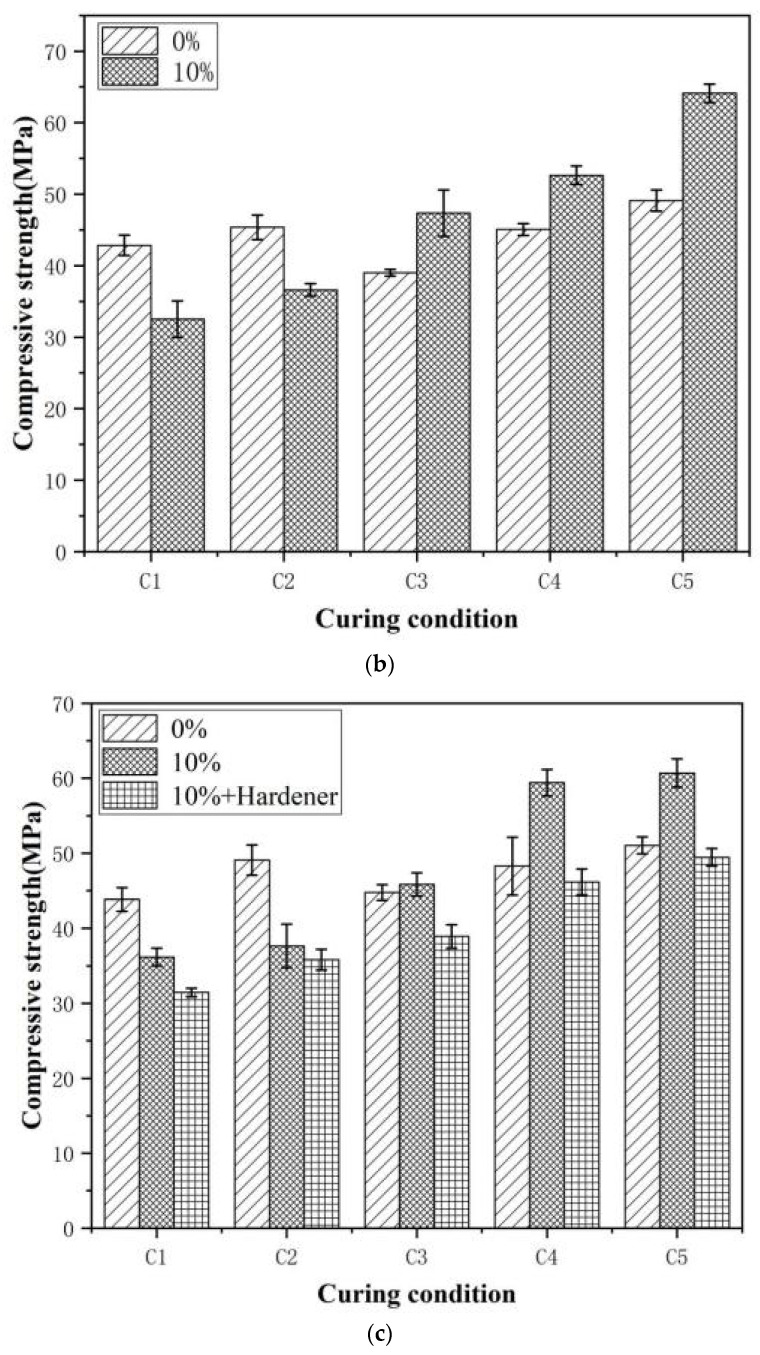
Compressive strength of mortar specimens under different curing conditions on Days (**a**) 14, (**b**) 21, and (**c**) 28.

The compressive strength of each specimen was tested on Days 14, 21, and 28, with the results shown in Figure 6.

Figure 6 shows that the curing conditions significantly affect the compressive strength of E-51-modified mortar. At 25 °C (55 ± 5% RH), 40 °C, and 60 °C, the strength of Sample is higher than that of Control 1 and increases with temperature. On Day 14 of curing at 60 °C (Group C5), the compressive strength of Sample reaches 61.6 MPa, which is 1.5-fold higher than that of Control 1. Subsequently, the strength growth slows, indicating that epoxy curing and cement hydration are essentially complete at an early age at 60 °C curing. On Day 28, the 40 °C cured group (Group C4) is close to that of Group C5, suggesting that at this temperature, epoxy curing is nearly completed within 28 d. At 25 °C (Group C3), the 14-day compressive strength was essentially comparable to that of the Control 1. At 28 days, the compressive strength increased by only 1.69%, indicating that epoxy curing requires a longer duration under the 25 °C condition.

Figure 6 shows that humidity influences strength development. In high-humidity environments (Groups C1 and C2), the strength of Sample at all ages is lower than that of Control 1. A decrease in humidity to 55 ± 5% RH (Group C3), the strength of the experimental group exceeds that of Control 1. This indicates that high humidity inhibits epoxy resin-based curing, while a low degree of curing adversely affects the mortar strength. Figure 6c shows that Control 2 exhibits lower Day 28 strength than the Sample-modified mortar and Control 1. This suggests that the external curing agent negatively impacts the mortar strength. This detrimental effect might outweigh the enhancement provided by epoxy curing. Water immersion (Group C1) further suppresses the reaction between E-51 and the curing agent in Control 2, resulting in the lowest strength.

In summary, in an alkaline environment of cement-based materials, E-51 can undergo endogenous curing without relying on external curing agents, which enhances the compressive strength of mortar. At 60 °C, curing requires 14 days; at 40 °C, it requires 28 days; at 25 °C, it requires even longer. High-humidity environments inhibit epoxy curing, resulting in reduced compressive strength of the mortar.

#### 3.2.2. Observing the Curing of Epoxy Resin in Cementitious Materials

**Figure 7:**
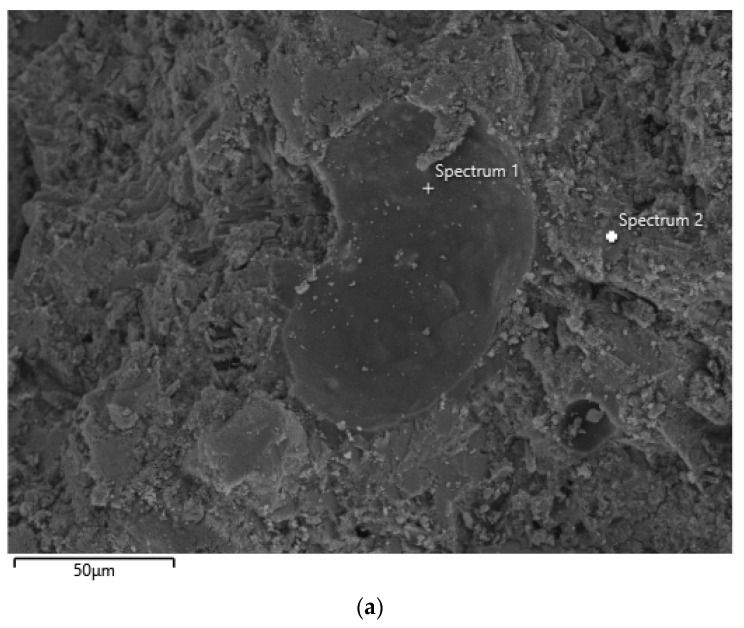

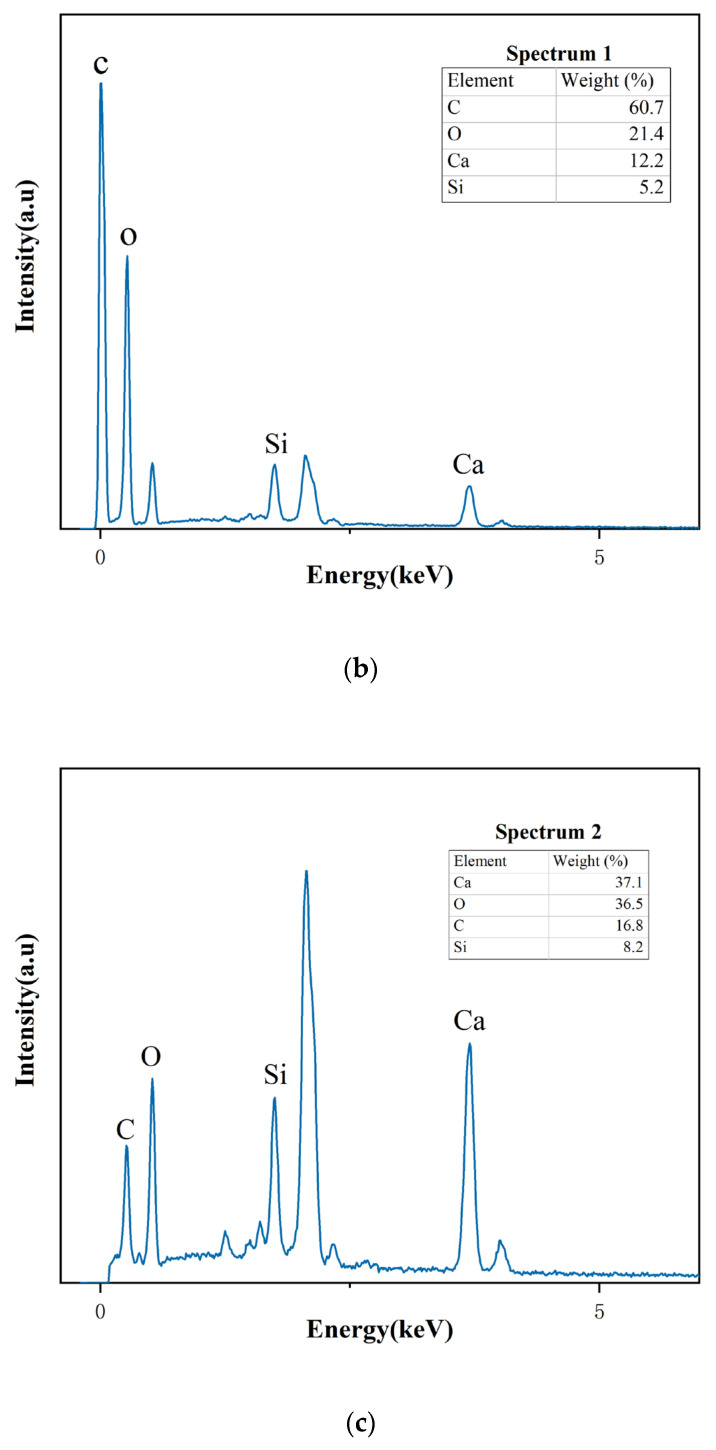

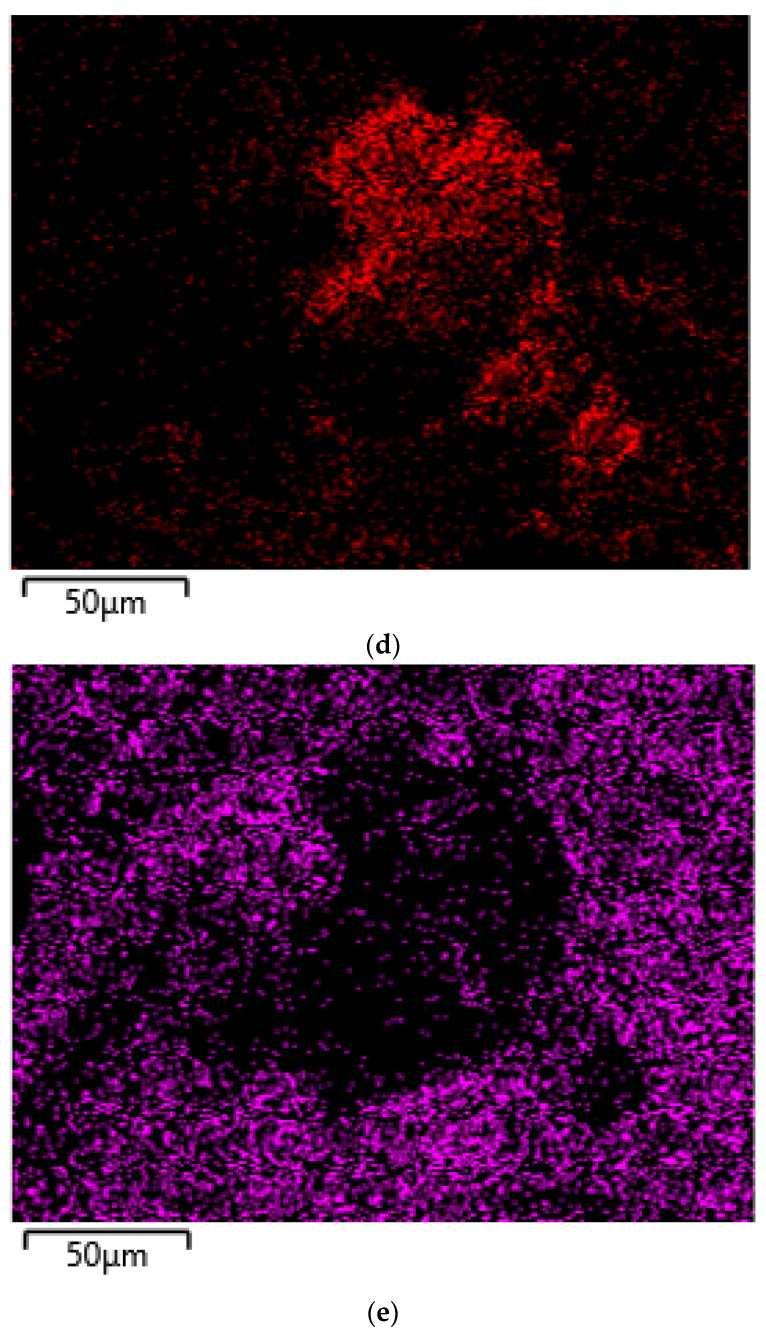
SEM image and EDS analysis of the cured epoxy resin in the cement-based matrix. (**a**) Morphology of the cured epoxy resin. (**b**,**c**) EDS point spectra (Spectrum 1 and 2). (**d**,**e**) EDS elemental mapping for carbon (C, shown in red) and calcium (Ca, shown in purple).

Epoxy resin E-51 and cement were mixed to form paste samples. After curing at 40 °C for 28 days and washing away the uncured epoxy resin with acetone, the samples were observed under SEM and analyzed for elemental distribution using EDS. The test results are shown in Figure 7.

The cured epoxy resin is observed in Figure 7. EDS analysis of Figure 7b,d indicates that the location marked as point 1 in Figure 7 corresponds to the cured epoxy.

After curing for 28 days at 25 °C, it is difficult to find cured epoxy. Analysis indicates that the low degree of curing resulted in most of the cured epoxy being washed away by acetone.

#### 3.2.3. Detection of Epoxy Curing in Mortar via EIS

**Figure 8:**
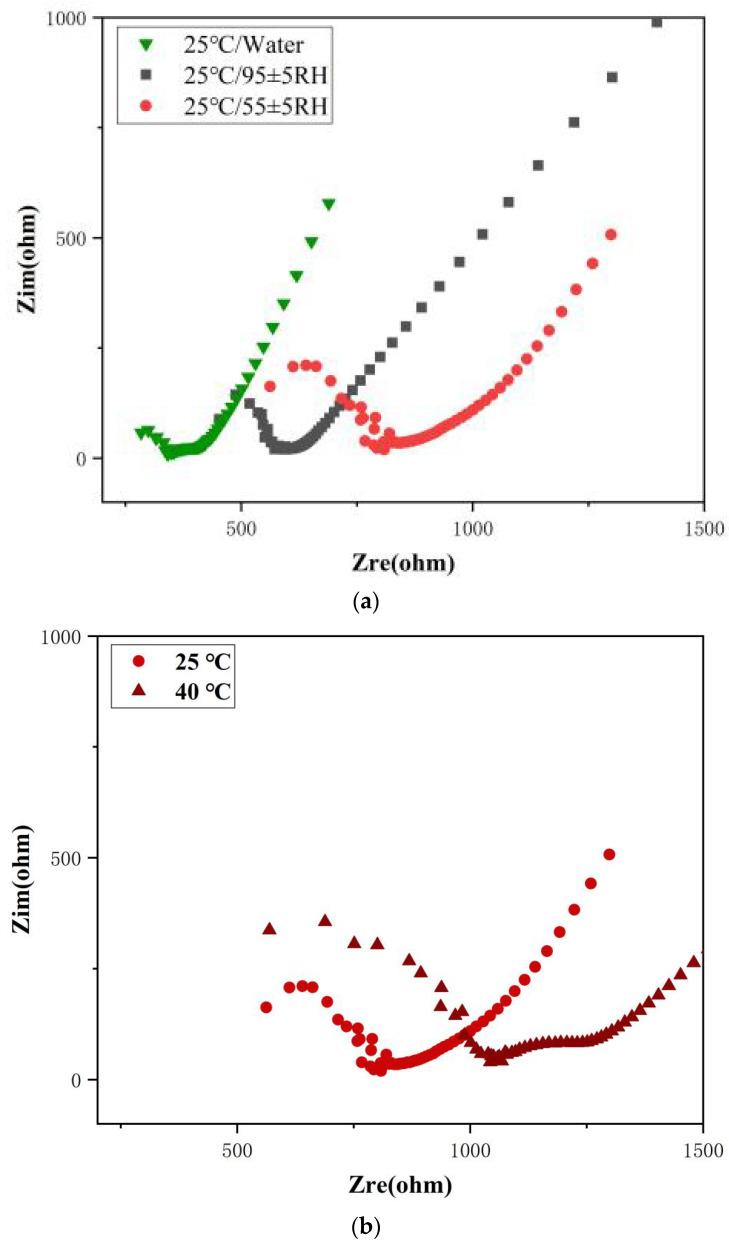

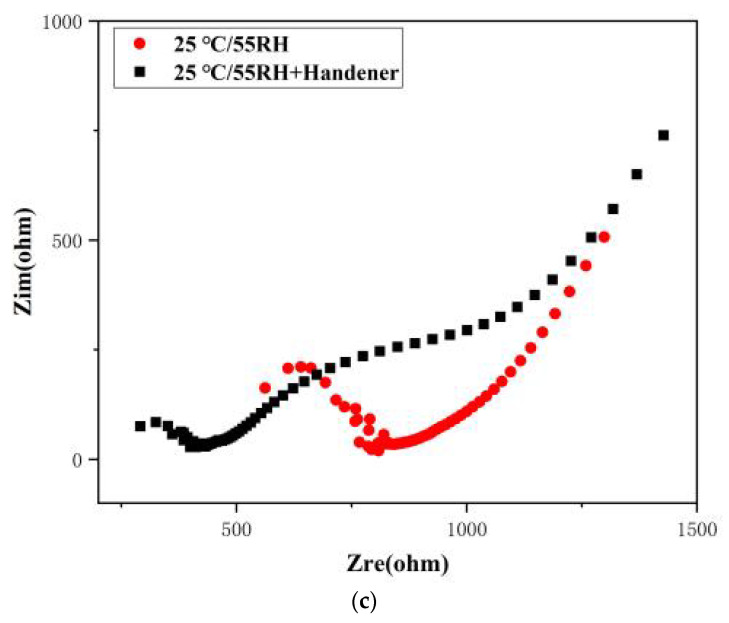
EIS plots of mortar specimens on Day 28 of curing under different conditions: (**a**) humidity levels, (**b**) temperatures, and (**c**) comparison with/without a curing agent.

EIS is utilized for qualitative analysis of epoxy curing degree in mortar. The impedance changes in the samples on Day 28 under different curing environments are presented in Figure 8.

The EIS spectra presented in Figure 8 are shown as Nyquist plots, each comprising a semicircle and straight line, characteristics of a Randles circuit. After curing, epoxy not only fills pores to form a three-dimensional network but also chemically bonds with the cement interface, impeding charge conduction and increasing impedance. This leads to an enlarged impedance radius. Therefore, a larger impedance radius in the Nyquist plot indicates a higher degree of epoxy curing.

From Figure 8a, it can be observed that as the humidity of the curing environment decreases, the impedance radius increases, indicating that the degree of epoxy curing inside the epoxy mortar increases with the reduction in environmental humidity. From Figure 8b, it can be observed that the impedance radius of the mortar cured at 40 °C is larger than that cured at 25 °C, suggesting that the degree of epoxy curing inside the mortar increases with rising temperature. The degree of epoxy curing has a positive effect on the compressive strength of the epoxy mortar, and the test results are consistent with the experimental findings shown in Figure 6.

It is worth noting that in Figure 8c, the impedance radius of the epoxy mortar with the curing agent is smaller than that without the curing agent. The analysis indicates that the amine-based curing agent introduces bubbles during mixing, leading to a decrease in the compactness of the mortar’s internal structure. Since the EIS test was conducted under high-humidity conditions, the reduced compactness of the sample results in a smaller impedance radius.

### 3.3. Application of Endogenous Epoxy Curing in Self-Healing Concrete

**Figure 9:**
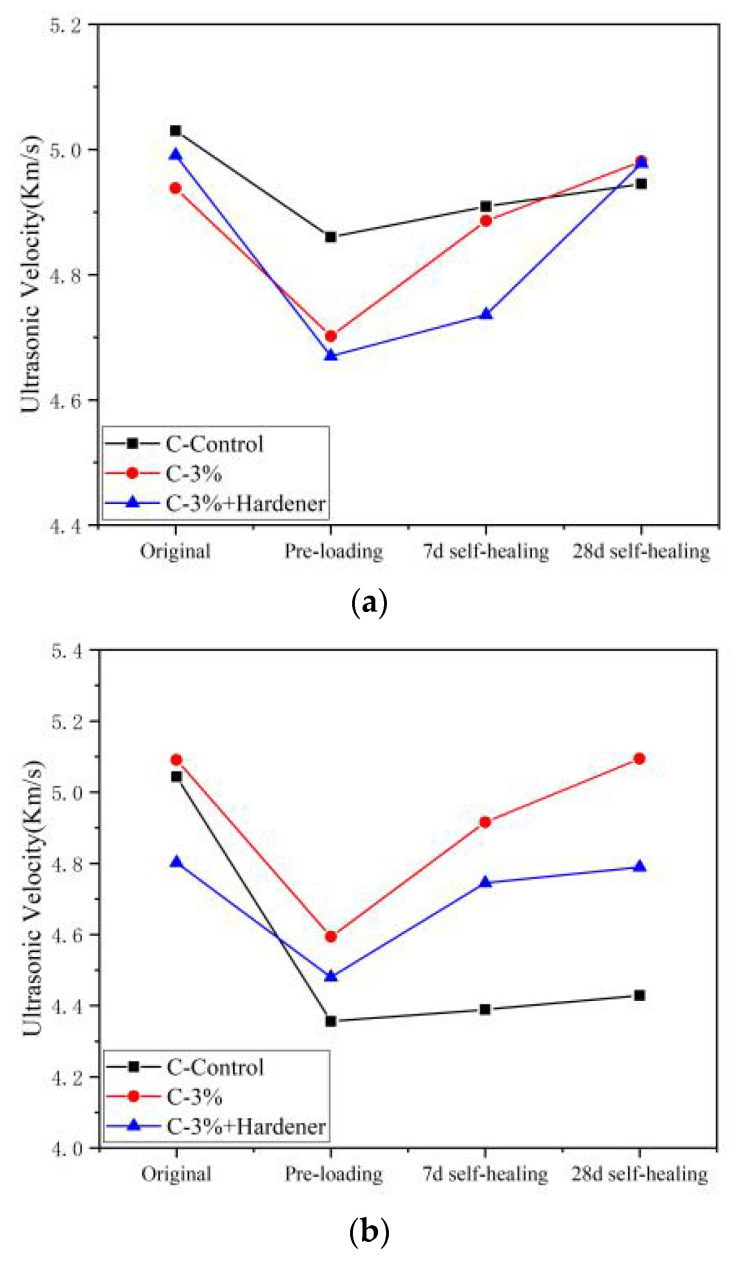
Curves of ultrasonic pulse velocity changes for the C-Control, C-3%, and (C-3% + Hardener): (**a**) 7- and (**b**) 28-day aging.

E-51 was encapsulated as a healing agent in capsules and incorporated into concrete to study the effect of its endogenous curing on self-healing performance. Three types of specimens were prepared: (i) a control group without capsules (C-Control), (ii) an experimental group containing 3 wt.% E-51 capsules (relative to the cement mass) (C-3%), and (iii) a control group with capsules and curing agent (C-3% + Hardener). The samples were cured at 25 °C and 95 ± 5% RH for 7 and 28 d and loaded to 60% of their compressive strength to induce damage, followed by natural healing in an indoor environment. The healing effectiveness was evaluated using the ultrasonic pulse velocity, and the results are presented in Figure 9.

Figure 9 shows that the ultrasonic pulse velocity of C-Control, C-3%, and (C-3% + Hardener) decreases significantly after loading damage, indicating the generation of internal cracks. During the healing period, the pulse velocity of C-3% and (C-3% + Hardener) recovers noticeably, returning nearly to the initial level after 28 d of healing. This suggests that the released E-51 flows and fills the cracks and cures them by bonding effectively.

The pulse velocity of C-Control aged for 7 d (Figure 9a) increases slightly with time after damage, which is attributed to continued hydration of incompletely reacted cement that partially heals the cracks. The recovery degree of C-3% and (C-3% + Hardener) is significantly higher than that of C-Control. Moreover, C-3% outperforms (C-3% + Hardener), demonstrating that in the alkaline environment of concrete, E-51 can undergo endogenous curing and achieve good healing effectiveness.

The cement hydration is largely complete for specimens aged for 28 d (Figure 9b), making it difficult for C-Control to achieve self-healing after damage. In contrast, the pulse velocity of C-3% and (C-3% + Hardener) recovers markedly during the healing period. C-3% returns to its initial pulse velocity after 28 d of healing, showing pronounced self-healing performance. E-51 cures the cracks rapidly in the early stage for C-3% + Hardener owing to the presence of the curing agent, completing healing within 7 d, after which pulse velocity becomes constant.

The recovery ratio of UPV (*R*) were calculated using Equation (2). The calculation results are presented in Table 7.

(2) $$R=\frac{v_{i}}{v_{0}}\times \;100\%$$

where $v_{0}$ is the initial wave velocity, $v_{i}$ is the wave velocity after healing.

Table 7 shows that concrete containing capsules exhibits considerable self-healing capacity after damage. After placing in an indoor environment for 28 d, the ultrasonic pulse velocity recovers to its initial value, with a *R* of 100%. Furthermore, the healing effect based on endogenous curing is superior to that of the system with an added curing agent.

When ultrasonic waves encounter defects such as voids or cracks during propagation, they undergo diffraction, leading to an increase in the propagation path—resulting in a reduction in ultrasonic wave velocity. Once the cracks are healed, the wave velocity recovers. However, since ultrasonic wave velocity is a relatively macroscopic parameter, its sensitivity to minor cracks is limited. Therefore, a full recovery of ultrasonic wave velocity to 100% can only indicate that most cracks have been healed. Relying solely on the Ultrasonic Pulse Velocity (UPV) as an indicator is insufficient for quantifying self-healing performance. In this study, the influence of crack width and crack morphology on ultrasonic wave velocity is not considered.

## 4. Conclusions

This study investigated alkali-catalyzed curing of E-51 and its endogenous curing behavior in cement-based materials. The main conclusions are mentioned below.

1. E-51 could achieve self-curing via alkali catalysis in an alkaline environment, but moisture significantly inhibited this reaction. At ambient temperature, the reaction was slow and required a prolonged curing time. Elevated temperature markedly accelerated the process, and curing could be completed within ~16 min at 60 °C.
2. In cement-based materials, E-51 could undergo endogenous curing without relying on external curing agents. Higher temperatures and lower humidity favored the curing reaction, enhancing the compressive strength of mortar. In contrast, high-humidity environments (e.g., water curing or standard curing) hindered curing, resulting in lower strength than the reference group. At 60 °C, the compressive strength of mortar containing 10 wt.% epoxy (by cement mass) was 1.5-fold higher than that of the control group after 14 d. At 40 °C, the 28 d compressive strength of epoxy-modified mortar was 1.2-fold higher than that of the control group and 1.3-fold higher than that of the group with an added curing agent. However, the compressive strength of epoxy mortar under high humidity conditions is not satisfactory. Meanwhile, at 25 °C, a longer curing time is required to achieve the effect of improving the compressive strength of the mortar.
3. Self-healing concrete based on endogenous epoxy curing exhibited excellent self-healing performance. After 28 d of self-healing, the *R* values of epoxy microcapsule-containing specimens aged 28 d were 100%. Moreover, the healing effect of the system without a curing agent was superior to that of the system with a curing agent.

The findings of this study provide a theoretical basis for the preparation of epoxy-modified mortar and self-healing concrete, avoiding the adverse effects associated with external curing agents and promoting their practical engineering application.

This study primarily focused on E-51 (epoxy value 0.48–0.54), which offered good fluidity and is widely used in concrete self-healing applications. The conclusions drawn are applicable to epoxy resins containing hydroxyl groups in their molecular chains (such as bisphenol-A type), while their applicability to other types of epoxy resins requires further verification.

## Figures and Tables

**Table 1:** Chemical and clinker mineral compositions of P·I 42.5 Portland cement used in the experiment.

Constitute	SiO_2_	Fe_2_O_3_	CaO	Mg	SO_3_	Na_2_O eq.	f-CaO	2CaO·SiO_2_	3CaO·SiO_2_	3CaO·Al_2_O_3_	4CaO·Al_2_O_3_·Fe_2_O_3_
Content/wt.%	22.15	3.12	64.76	2.98	0.65	0.57	0.90	19.01	59.42	6.36	9.48

Notes: Sodium oxide equivalent (Na_2_O eq.) is the total alkali content in Portland cement, expressed as Na_2_O. Free calcium oxide (f-CaO) refers to the unreacted CaO present in Portland cement clinker.

**Table 2:** ISO standard sand particle diameter distribution used in the experiment.

Square Hole Side Length (mm)	Cumulative Screening Margin (%)	Square Hole Side Length (mm)	Cumulative Screening Margin (%)
2.0	0	0.5	67 ± 5
1.6	7 ± 5	0.16	86 ± 5
1.0	33 ± 5	0.08	99 ± 5

**Table 3:** Major chemical composition and mineral composition of the S95 mineral powder.

Constitute	SiO_2_	Al_2_O_3_	CaO	MgO	SO_3_
Content (wt.%)	33.06	15.04	39.29	9.96	1.9

**Table 4:** Chemical and mineral compositions of Fly ash.

Property	Al_2_O_3_	SiO_2_	Water Content	Cl^−^	SO_3_	CaO	Alkali Content	Fe Content
Test Result/wt%	24.2	45.1	0.85	0.015	2.1	5.6	1.2	0.85

**Table 5:** Proportion of concrete specimens.

Sample	Material Dosage (kg m^−3^)						Hardener	Micro Capsules (%)
	Water	Cement	Fly Ash	Slag Powder	Sand	Aggregate		
C-Control	160	369	55	138	610	1087	0	0
C-3%	160	369	55	138	610	1087	0	3
C-3% + Hardener	160	369	55	138	610	1087	10% of the microcapsule mass	3

**Table 6:** Reaction enthalpy and relative curing degree of E-51–NaOH-p measured via DSC.

Storage Temperature (°C)	Curing Time (d)	Reaction Enthalpy (J mg^−1^)	Relative Curing Degree (%)
25	0	2.64	0
	7	2.27	13.70
	14	1.70	35.67
	21	1.18	55.22
	28	0.50	81.04
40	0	2.66	0
	7	1.04	60.88
	14	1.65	93.80
	21	0.73	97.26

**Table 7:** Calculated *R* of the concrete specimens.

Age (d)	Test Group	*R* (%)	
		7 d Self-Healing	28 d Self-Healing
7	C-Control	97.59	98.31
	C-3%	98.95	100.87
	C-3% + Hardener	94.89	99.74
28	C-Control	87.01	87.8
	C-3%	96.56	100.06
	C-3% + Hardener	98.81	99.73

## Data Availability

The original contributions presented in this study are included in the article. Further inquiries can be directed to the corresponding author.
